# Bacterial-like inflammatory response in children with adenovirus leads to inappropriate antibiotic use: a multicenter cohort study

**DOI:** 10.1007/s15010-024-02405-8

**Published:** 2024-10-08

**Authors:** Cristina Moracas, Marco Poeta, Francesca Grieco, Agnese Tamborino, Maria Moriondo, Marta Stracuzzi, Alfredo Diana, Laura Petrarca, Simona Marra, Amelia Licari, Stefano Linsalata, Chiara Albano, Anna Condemi, Ester Del Tufo, Teresa Di Fraia, Liana Punzi, Eleonora Ardia, Andrea Lo Vecchio, Eugenia Bruzzese, Claudia Colomba, Vania Giacomet, Fabio Midulla, Gian Luigi Marseglia, Luisa Galli, Alfredo Guarino

**Affiliations:** 1 Pediatric Infectious Disease Unit, Department of Maternal and Child health, University Hospital “Federico II”, Naples, 80131 Italy; 2 PhD National Programme in One Health approaches to infectious diseases and life science research, Department of Public Health, Experimental and Forensic Medicine, University of Pavia, Pavia, 27100 Italy; 3 Department of Translational Medical Science, University of Naples “Federico II”, Naples, 80131 Italy; 4 Infectious Disease Unit, Meyer Children’s Hospital IRCCS, Florence, 50139 Italy; 5 Immunology Division, Section of Pediatrics, Meyer Children’s Hospital IRCCS, Florence, 50139 Italy; 6 Department of Pediatrics, Pediatric Infectious Disease Unit, L. Sacco Hospital, University of Milan, Milan, 20157 Italy; 7 Department of Maternal, Infantile and Urological Sciences, Sapienza University of Rome, Rome, 00185 Italy; 8 Pediatric Unit, Department of Clinical, Surgical, Diagnostic and Pediatric Sciences, University of Pavia, Pavia, 27100 Italy; 9 Pediatric Clinic, Fondazione IRCCS Policlinico San Matteo, Pavia, 27100 Italy; 10 Department of Health Promotion, Mother and Child Care, Internal Medicine and Medical Specialties-University of Palermo, Palermo, 90127 Italy; 11 Department of Pediatrics, S. Maria delle Grazie Pozzuoli Hospital, Naples, 80078 Italy; 12 Pediatric Unit, Department of Women’s and Children’s Health, S. Maria della Speranza Hospital of Battipaglia, Salerno, 84091 Italy; 13 Department of Health Sciences, University of Florence Infectious Disease Unit Meyer Children’s Hospital IRCCS, Florence, 50139 Italy

**Keywords:** Viruses, Respiratory infection, Hyperinflammation, Molecular biology, Biomarkers

## Abstract

**Purpose:**

To compare the clinical severity of Human Adenovirus (HAdV) infection with other viral diseases in a cohort of children, evaluating presentation, therapy, and outcome.

**Methods:**

We conducted a retrospective multicenter cohort study in Italian children hospitalized from January to December 2023 for respiratory symptoms. The study included children with HAdV infection presenting primarily with respiratory symptoms. Patients with isolated gastrointestinal involvement or coinfection with bacteria were excluded.

**Results:**

A total of 171 children were enrolled: 98 with HAdV infection (age 44.3 ± 37.9 months) and 73 with other viruses (age 20.4 ± 27.2 months). In the first group, 57.1% had a coinfection with one or more additional viruses. The most common symptoms were fever (89.8%), cough (73.5%) and sore throat (52%). Respiratory distress and hypoxemia were more frequent in the non-HAdV group. Children with HAdV infection demonstrated significantly higher C-reactive protein levels (50.8 ± 54.2 vs. 16.5 ± 33.8 mg/L, *p* < 0.001), experienced a longer duration of fever (4.9 ± 3.6 vs. 3.4 ± 2.3 days, *p* = 0.009) and were more likely to receive antibiotic treatment (77.6% vs. 27.4%, *p* < 0.001). No differences were observed in hospitalization stay, rate of complications, and ICU admission.

**Conclusions:**

Interestingly, our data suggests that HAdV-infected children exhibit a more pronounced inflammatory response despite experiencing less severe respiratory symptoms compared to other viruses. The presence of prolonged fever and a strong inflammatory response often leads to antibiotic overuse during the initial phase, when the viral etiology is yet to be confirmed. Early and accurate identification of HAdV infection is crucial to optimize treatment strategies and minimize unnecessary antibiotic use.

## Graphical abstract

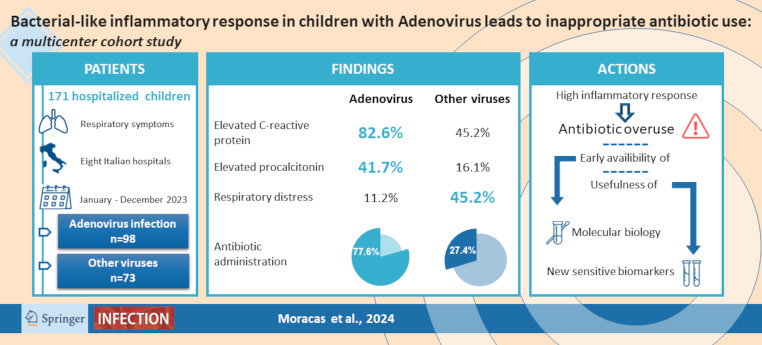

## Introduction

Respiratory tract infections are a significant cause of morbidity and healthcare utilization in the pediatric population. Human Adenovirus (HAdV) is particularly important among these infections because of its clinical manifestations and potentially severe course [1]. HAdV is a non-envelope and double-stranded DNA virus capable of producing a spectrum of diseases ranging from mild upper respiratory tract symptoms to severe lower respiratory tract infections, such as pneumonia and bronchiolitis [2].

Transmission of HAdV occurs via aerosolized droplets or direct conjunctival inoculation, but the fecal-oral route or contact with contaminated fomites is also possible [3]. The incubation period varies from 2 days to 2 weeks, depending on the serotype and the route of transmission [4].

HAdV may cause epidemics of febrile respiratory illness in children attending schools and daycare centers [5]. In fact, due to a lack of humoral immunity, more than 80% of infections occur in children below four years of age, and HAdV is responsible for up to 10% of respiratory illnesses in this age group [1]. In addition to respiratory infections, HAdV causes various diseases, including conjunctivitis, gastroenteritis, and hemorrhagic cystitis [2].

HAdV infections are frequently mild and self-limiting; however, life-threatening conditions can occur in younger children and immunocompromised patients, who are prone to develop disseminated infections, severe multiorgan damage, and more rare manifestations such as meningitis, encephalitis, myocarditis, pulmonary dysplasia, intestinal intussusception, and sudden infant death [1]. Furthermore, patients with severe pneumonia have up to 30% risk of developing long-term respiratory effects, such as post-infectious bronchiolitis obliterans and bronchiectasis [6].

Without appropriate diagnostic tests based on nucleic acid detection, the differential diagnosis of HAdV infection may be difficult. During HAdV infections, increased C-reactive protein (CRP) and procalcitonin, white blood cells, and neutrophilic shift [7] may mimic bacterial infections. Furthermore, Kawasaki disease, Multisystem Inflammatory Syndrome of children (MIS-C), or other hyperinflammatory syndromes may share with HAdV clinical features such as prolonged fever, skin rash, and adenopathy. For these reasons, changes in laboratory parameters and persistence of symptoms, even in self-limiting infections, increase the concern of parents and physicians, leading to unnecessary hospitalizations and treatments (e.g., antibiotics, steroids, immunoglobulins) [7].

Reports of HAdV infections in hospitalized children are limited, as only a few studies have been published, and severity outcomes are unclear. This study aimed to characterize pediatric HAdV infections, evaluating clinical presentations, biochemical markers, and radiological findings and investigating potential factors associated with disease severity, including age, underlying medical conditions (comorbidities), and co-infections, compared with other common respiratory viruses.

## Patients and methods

### Study design and population

We conducted a retrospective multicenter cohort study in children aged 0–17 years with clinical evidence of acute respiratory infections admitted to 8 hospitals between January and December 2023. The hospitals included referral and non-referral urban centers throughout Italy. Considering that hospitalized children are certainly those with more severe symptoms, findings of our study are limited to patients with moderate or severe diseases.

**Fig. 1:**
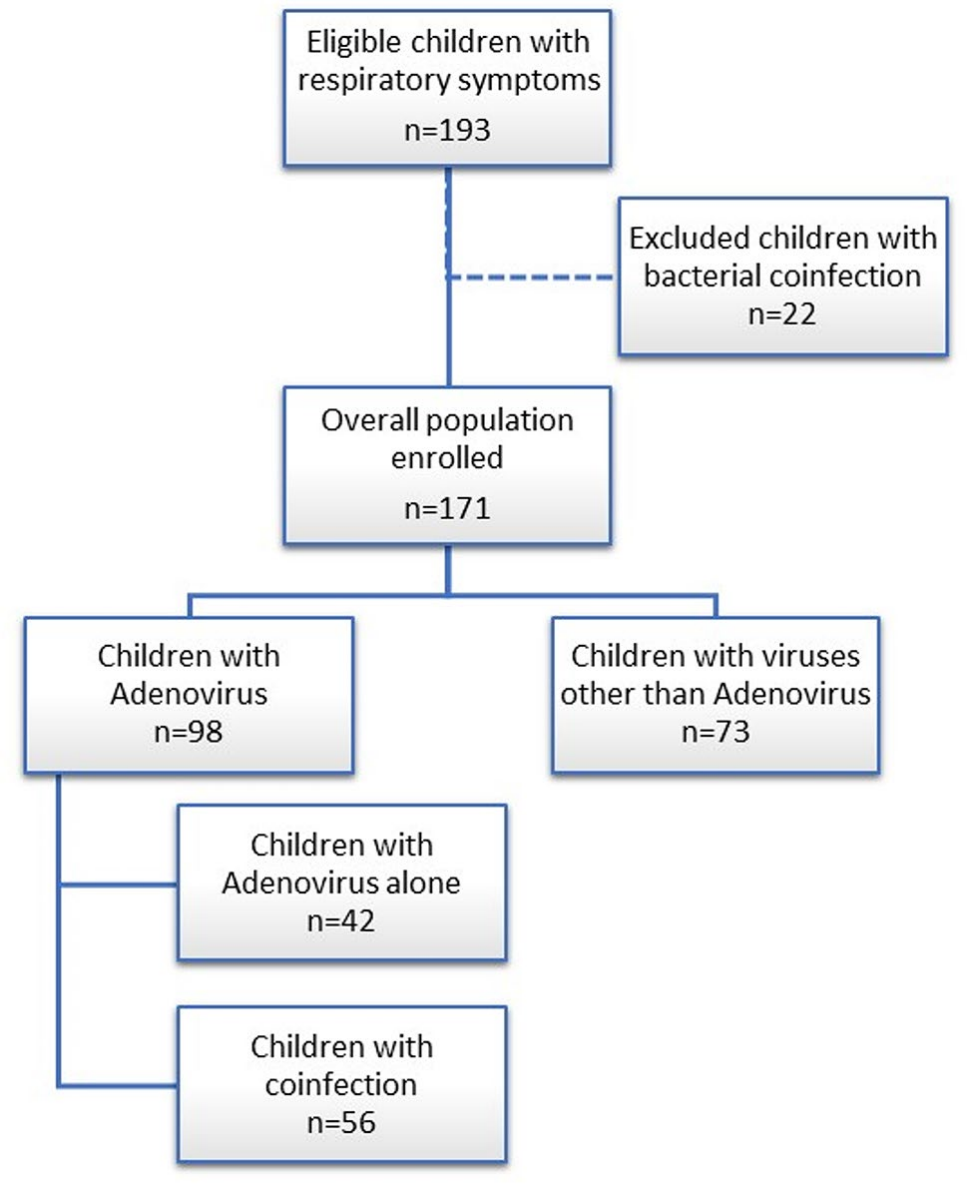
Study population. Eligible hospitalized children with respiratory symptoms due to Human Adenovirus or other viruses

The study design is illustrated in Fig. 1. Children with upper (e.g. rhinorrhea, cough, sore throat) and/or lower respiratory tract signs and symptoms (e.g., a high respiratory rate for the patient’s age, use of accessory respiratory muscles, intercostal retractions, nasal flaring, crackles or wheezing, and low oxygen saturation levels) were enrolled and categorized based on nasopharyngeal swab detection of HAdV, assessed by Real Time-Polymerase Chain Reaction (RT-PCR).

The study group included children hospitalized with at least one HAdV-positive respiratory sample during the study period. Children with documented HAdV infection but only GI involvement were excluded from the study. Children with bacterial infections (such as urinary tract infections, sepsis, or GI infections) detected by bacterial cultures and children with documented bacterial pneumonia (Mycoplasma pneumoniae, Chlamydia pneumoniae, Streptococcus pneumoniae, or others) were excluded. For all patients enrolled in the study, we assumed that bacterial coinfections were unlikely for the absent response to antibiotics or for spontaneous resolution without antibiotic therapy.

The control group included both children with a diagnosis of other respiratory viral infections (SARS-CoV-2, Respiratory Syncytial Virus, rhinovirus, parainfluenza, influenza A-B, bocavirus, enterovirus) and with respiratory symptoms but negative for HAdV.

To define the specific role of HAdV in our outcomes, children with HAdV infection were classified into two groups based on the presence or absence of other respiratory viral agents (coinfection group) and further analyzed.

### Data collection and outcome measures

For each patient, the following data were collected and recorded in an anonymized dataset: demographics, presence of comorbidities, clinical presentations, laboratory tests, chest X-ray, treatments, outcomes, and time to discharge. All children enrolled performed laboratory tests including complete blood count, but not all of them underwent x-rays. Regarding laboratory tests, if performed more than one time during the entire hospitalization, we only considered the worst value detected (highest or lowest depending on the parameter).

The comparative outcomes in children with HAdV infection and children with other respiratory viral infections included fever duration, inflammatory indexes elevation, length of hospitalization, use of antimicrobial therapy, need for oxygen and corticosteroids therapy, rate of complications, and intensive care unit (ICU) admission. Finally, to evaluate the real role of HAdV and to exclude the contribute of viral coinfections in shaping the clinical and biochemical presentation in the HAdV group, we assessed the same outcomes both between children with HAdV alone versus those with viral co-infections (HAdV plus any other viral respiratory disease), and between children with HAdV alone versus those with other viral infections.

The study was conducted in accordance with the Declaration of Helsinki and approved by the Ethics Committee for Biomedical Activities, University of Naples Federico II, Naples, Italy (Protocol number 76/2023). Written informed consent to use clinical data was obtained from the parents of the children involved in the study.

### Statistical analysis

Statistical analyses were performed with IBM SPSS Statistics for Windows, Version 29.0 (Armonk, NY, USA: IBM Corp). Continuous variables were reported as mean and Standard Deviation (SD), or median and InterQuartile Range (IQR), according to their distribution, and compared using a t-test or Mann–Whitney test, as appropriate. Categorical variables expressed as frequencies and percentages were compared using Fisher’s exact test or χ^2^. Univariable logistic regression analysis was used to investigate the variables associated with infection, with risk expressed as Odds Ratio (OR) with 95% confidence intervals (CI). Pearson’s correlation coefficient was used to measure the strength of association between variables. The level of significance was set at 0.05 (2-tailed).

## Results

### Differences in demographics and baseline conditions between children with HAdV and other viral infections

A total of 193 hospitalized children with a diagnosis of acute respiratory infection were initially included in the study. Twenty-two patients with a bacterial coinfection were excluded, while the remaining population of 171 children was further analyzed (95 males, 55.6%, mean age 34.1 ± 35.7 months).

**Fig. 2:**
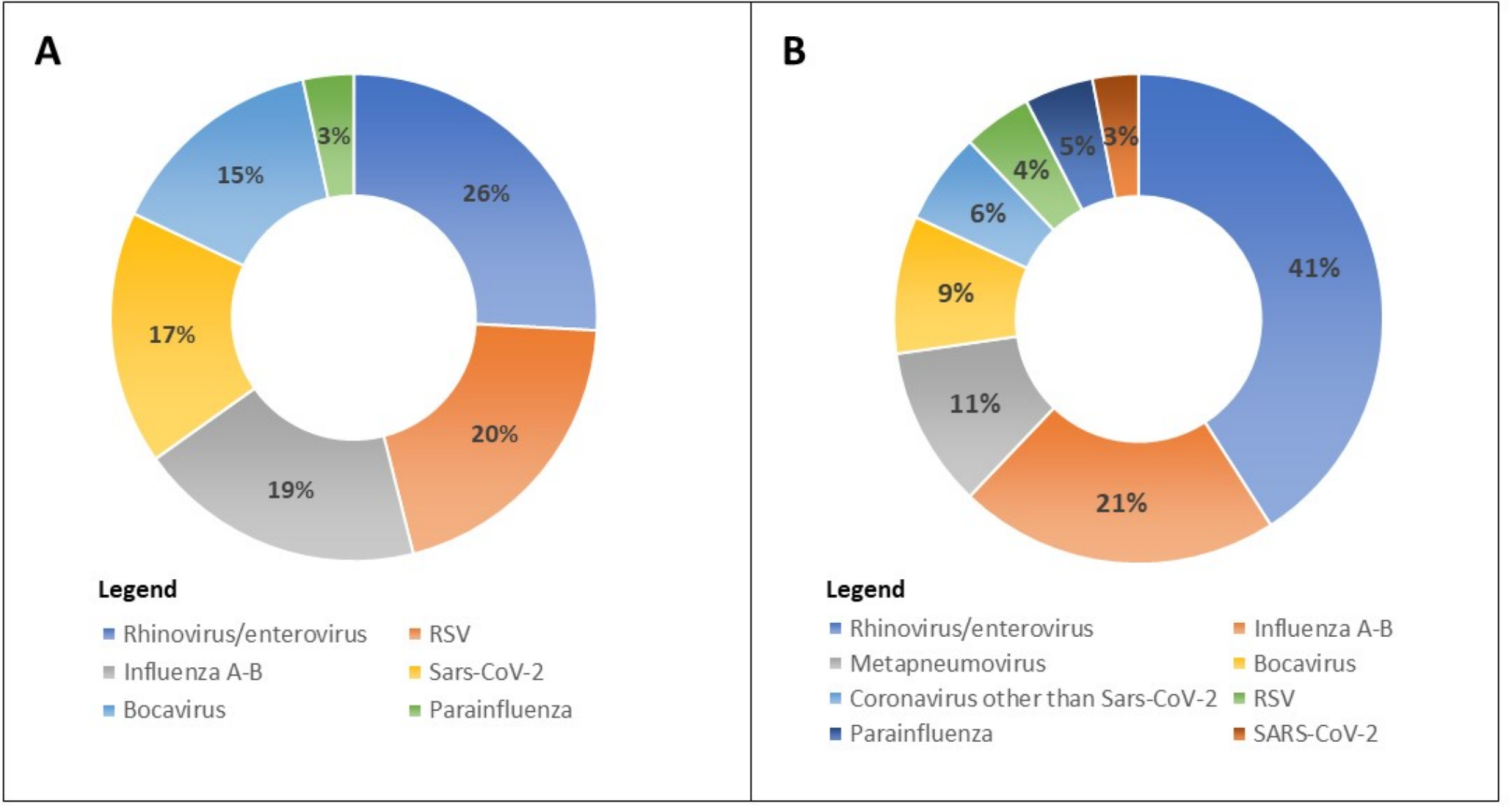
(**a**) Frequency of specific viruses identified in children diagnosed with respiratory viral infections other than HAdV (**b**) Distribution of viral coinfections in children with HAdV. Abbreviations: HAdV, Human Adenovirus; RSV, Respiratory Syncytial Virus

Among them, 98/171 children (57.3%) had HAdV infection (mean age 44.3 ± 37.9 months), and 73/171 (42.6%) had a diagnosis of respiratory viral infection other than HAdV (mean age 20.4 ± 27.2 months). The etiology of non-HAdV agents is reported in Fig. 2a. Most HAdV-positive cases were detected among children < 5 years of age (75.5%, 74/98). Regarding underlying conditions and past medical history, 20 (11.7%) children of the overall population had an underlying chronic disease, such as epilepsy, asthma, autism, Klinefelter syndrome, kidney abnormalities, or leukemia, with a higher frequency in the non-HAdV group compared with the HAdV-group (19.2% vs. 6.1%, *p* = 0.009).

In our cohort, HAdV infection was detected throughout the year; however, two outbreaks were identified in March-May 2023 and October-December 2023.

### Clinical features

The clinical features of enrolled children are summarized in Table 1. Fever was the main clinical manifestation in both groups, being more common in children with HAdV (89.8% versus 74.0%, *p* = 0.006), who also had a longer fever duration (4.9 ± 3.6 versus 3.4 ± 2.3 days, *p* = 0.009) compared with the control group. Children with HAdV more commonly reported sore throat (52.0% versus 13.7%, *p* < 0.001) and lymphadenopathy (31.6% versus 12.3%, *p* = 0.02) than HAdV-negative children who, in turn, had more severe respiratory symptoms, including distress (45.2% versus 11.2%, *p* < 0.001) and lower oxygen saturation level (96.0 ± 3.8 versus 98.0 ± 1.8, *p* < 0.001), with a higher number of children with hypoxemia ≤ 94% (23.3% vs. 3.1%, *p* < 0.001). Results were also confirmed if children with comorbidity were excluded from the analysis. Febrile seizures were detected in 15 of 98 (15.3%) patients with HAdV infection and in 5 of 73 (6.8%) children with other illnesses, although this difference did not reach statistical significance (*p* = 0.07). The incidence of other symptoms (cough, conjunctivitis, GI symptoms, and skin involvement) did not differ between groups. In HAdV patients, skin rashes included maculo-papules, papules, and erythema. Four children with HAdV (4.1%) presented with a clinical picture meeting the criteria of Kawasaki syndrome, and intravenous Immunoglobulins were successful administered in 2 cases.

**Table 1:** Demographic and clinical features of the overall population, including children with HAdV and children with other respiratory infections; *p* refers to HAdV versus other infections. Abbreviations: HAdV, Human adenovirus; n, number; SD, standard deviation

	Overall population (*n* = 171)	HAdV (*n* = 98)	Other viruses (*n* = 73)	*p*
Demographic features				
Male sex, *n* (%)	95 (55.6)	59 (60.2)	36 (49.3)	0.161
Mean age, months (SD)	34.1 (35.7)	44.3 (37.9)	20.4 (27.2)	**< 0.001**
At least one comorbidity, *n* (%)	20 (11.7)	6 (6.1)	14 (19.2)	**0.009**
At least one coinfection, *n* (%)	93 (54.3)	56 (57.1)	20 (27.4)	**< 0.001**
Clinical features				
Fever, *n* (%)	142 (83.0)	88 (89.8)	54 (74.0)	**0.006**
Peak body temperature, ◦C mean (SD)	38.8 (1.3)	38.7 (1.5)	38.7 (0.8)	0.950
Fever duration, days (SD)	4.4 (3.28)	4.9 (3.6)	3.4 (2.3)	**0.009**
Cough, *n* (%)	129 (75.4)	72 (73.5)	57 (78.1)	0.305
Respiratory distress, *n* (%)	44 (25.7)	11 (11.2)	33 (45.2)	**< 0.001**
SpO2, % (SD)	36 (21.7)	98.0 (1.8)	96.0 (3.8)	**< 0.001**
Wheezing, *n* (%)	33 (19.3)	16 (16.3)	17 (23.3)	0.172
Sore throat, *n* (%)	61 (35.7)	51 (52.0)	10 (13.7)	**< 0.001**
Conjunctivitis, *n* (%)	16 (9.4)	10 (10.2)	6 (8.2)	0.435
Vomiting, *n* (%)	25 (14.6)	15 (15.3)	10 (13.7)	0.473
Diarrhea, *n* (%)	21 (12.3)	14 (14.3)	7 (9.6)	0.247
Abdominal pain, *n* (%)	16 (9.4)	10 (10.8)	6 (8.2)	0.435
Rash, *n* (%)	19 (11.1)	13 (13.3)	6 (8.2)	0.215
Febrile seizures, *n* (%)	20 (11.7)	15 (15.3)	5 (6.8)	**0.07**
Lymphadenopathy, *n* (%)	40 (23.4)	31 (31.6)	9 (12.3)	**0.02**
Length of hospital stay, mean (SD)	4.9 (3.0)	5.1 (3.1)	4.8 (2.8)	0.510

### Laboratory and radiological findings

Table 2 summarizes the biochemical and radiological findings. Children with HAdV showed increased inflammatory markers compared to children with other respiratory infections, as demonstrated by mean CRP (50.8 ± 54.2 versus 16.5 ± 33.8 mg/L, *p* < 0.001), procalcitonin (1.9 ± 3.3 versus 0.7 ± 1.2 ng/mL, *p* = 0.047) and fibrinogen levels (461.3 ± 126.3 versus 353.2 ± 137.0 mg/dl, *p* = 0.007). Of the 98 children with HAdV, 81 (82.6%) had elevated CRP (> 5 mg/l) compared to 33 (45.2%) children with other respiratory virus infections (*p* < 0.001). Procalcitonin, performed for 140 children, was increased (> 0.5 ng/ml) in 35 patients (41.7%) with HAdV infection and in 9 (16.1%) with other respiratory viruses (*p* = 0.001). Leucocyte count was slightly higher in children with HAdV (13572 ± 7541 versus 11161 ± 4908 cells/mm3, *p* = 0.020) and was associated with higher neutrophil (8691 ± 6792 versus 5349 ± 4003 cells/mm^3^, *p* < 0.001), and monocyte counts (1369 ± 677 versus 972 ± 592 cells/mm^3^, *p* < 0.001). No anemia and thrombocytosis were noted in both groups. Liver enzymes were within the normal ranges in almost all patients. Within the group of HAdV-children, comparing patients with upper versus those with lower respiratory infections, no difference was found in inflammatory response, in terms of procalcitonin, neutrophil and lymphocyte count and fibrinogen levels. CRP value was slightly higher in children with lower respiratory tract infection although this difference did not reach statistical significance (46.6 ± 53.5 versus 55.7 ± 55.2 mg/L, *p* = 0.753).

**Table 2:** Biochemical and radiological findings of the overall population, including children with HAdV and children with other respiratory infections; *p* refers to HAdV versus other infections. Abbreviations: HAdV, Human adenovirus; n, number; SD, Standard Deviation; WBC, White Blood cells; CRP, C-reactive protein; AST, aspartate aminotransferase; ALT, alanine aminotransferase

	Overall population (*n* = 171)	HAdV (*n* = 98)	Other viruses (*n* = 73)	*p*
Biochemical parameters, means (SD)				
Haemoglobin, g/dl	11.7 (1.2)	11.9 (1.1)	11.4 (1.3)	**0.004**
WBC, cells/µL	12547 (6643)	13572 (7541)	11161 (4908)	**0.020**
Neutrophils, cells/µL	7270 (5991)	8691 (6792)	5349 (4003)	**< 0.001**
Lymphocytes, cells/µL	3986 (2572)	3630 (2363)	4467 (2775)	**0.037**
Monocytes, cells/ µL	1185 (667)	1369 (677)	972 (592)	**< 0.001**
Platelet count, x10^3^ cells/µL	359.8 (133)	353.9 (116)	367.8 (153)	0.505
CRP, mg/L	36.7 (49.7)	50.8 (54.2)	16.5 (33.8)	**< 0.001**
Procalcitonin, ng/mL	1.5 (2.9)	1.9 (3.3)	0.7 (1.2)	**0.047**
Fibrinogen, mg/dl	418.5 (139.8)	461.3 (126.3)	353.2 (137.0)	**0.007**
D-Dimer, ng/mL	1347 (1890)	1290 (2457)	1415 (985.3)	0.877
AST, U/L	49.4 (65.3)	37.7 (14.9)	68.3 (101.8)	**0.007**
ALT, U/L	32.6 (57.7)	20.3 (17.7)	49.6 (84.5)	**0.002**
Creatine kinase, U/L	117.1 (116.4)	90.7 (58.2)	144.4 (148.1)	**0.048**
Chest X-ray, *n* (%)				
Abnormal findings	55/84 (65.5)	35/58 (60.3)	20/26 (76.9)	0.154
Interstitial	20/84 (23.8)	13/58 (22.4)	7/26 (26.9)	0.653
Lobar	35/84 (41.7)	22/58 (37.9)	13/26 (50.0)	0.299

Chest radiography was performed in 58 HAdV children, of which 35 showed abnormal findings (60.3%). Multiple- or single-lobar/segment consolidations were the most common finding (22/58 children, 37.9%), followed by interstitial involvement (13/58 children, 22.4%). No difference in the frequency of radiographic abnormalities was found between HAdV and non-HAdV children.

### Treatment and outcomes

A total of 24.5% of children with HAdV infection and 17.8% of children with other infections received systemic steroids (*p* = 0.195), whereas 42 (42.9%) children in the HAdV group and 15 (20.5%) in the control group received inhaled steroids (*p* = 0.002). The number of children who required oxygen supplementation was significantly higher in the control group (37.0% versus 9.2%, *p* < 0.001) (Table 3).

**Table 3:** Therapies and outcomes of the overall population, including children with HAdV and children with other respiratory infections; *p* refers to HAdV versus other infections. Abbreviations: HAdV, Human adenovirus; n, number; SD, standard deviation; NA, not applicable

	Overall population (*n* = 171)	HAdV (*n* = 98)	Other viruses (*n* = 73)	*p*
Therapies				
Parenteral rehydration, *n* (%)	69 (40.4)	36 (36.7)	33 (45.2)	0.182
Antibiotics, *n* (%)	96 (56.1)	76 (77.6)	20 (27.4)	**< 0.001**
Antibiotic duration, mean (SD)	5.5 (2.3)	5.2 (2.1)	6.7 (2.9)	**0.022**
Systemic corticosteroids, *n* (%)	37 (21.6)	24 (24.5)	13 (17.8)	0.195
Bronchodilators, *n* (%)	62 (36.3)	38 (38.8)	24 (32.9)	0.264
Inhaled steroids, *n* (%)	57 (33.3)	42 (42.9)	15 (20.5)	**0.002**
Oxygen supplementation, *n* (%)	36 (21.1)	9 (9.2)	27 (37.0)	**< 0.001**
Intravenous Immunoglobulin, *n* (%)	2 (1.2)	2 (2.0)	0 (0)	0.327
Outcome				
Complications, *n* (%)	11 (6.4)	5 (5.1)	6 (8.2)	0.304
Death, *n* (%)	0 (0)	0 (0)	0 (0)	NA
Intensive care, *n* (%)	5 (2.9)	1 (1.1)	4 (5.5)	0.118

**Fig. 3:**
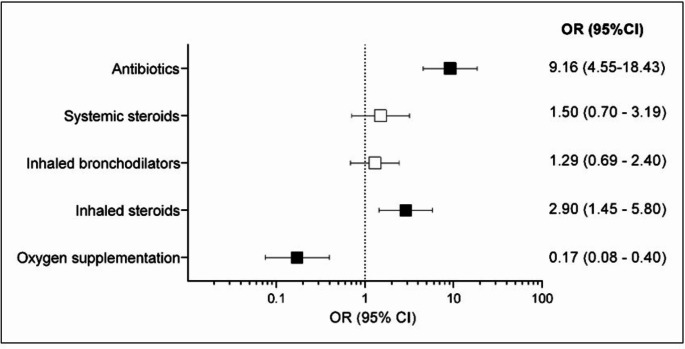
Therapeutic interventions in children with respiratory symptoms due to HAdV and other viruses. Black squares indicate significant results; white squares indicate nonsignificant results. Abbreviations: OR, odds ratio; CI, confidence interval

Children with HAdV infection were more frequently treated with an antibiotic than those with other infections, with a 9-fold increased probability of being treated in the HAdV group (OR 9.16, 95%CI 4.55–18.43) (Fig. 3). The most frequently antibiotics utilized in the HAdV cohort were ceftriaxone (35.1%), amoxicillin or amoxicillin/clavulanic acid (29.8%), the association of ceftriaxone and macrolide (15.8%) or the macrolide alone (15.8%), with poor clinical improvement within 24–48 h. In 14/76 children (18.4%) the antibiotic was stopped within 72 h, when the positive result of the adenovirus test became available.

Children with HAdV and those with other infections did not show differences in the length of hospital stay and complication rates (*p* > 0.05). Few children needed intensive care, and, although a higher proportion of patients with other infections were admitted to ICU (5.5% versus 1.1%), the difference did not reach statistical significance (*p* = 0.118). Deaths were not reported in either group (Table 3).

**Fig. 4:**
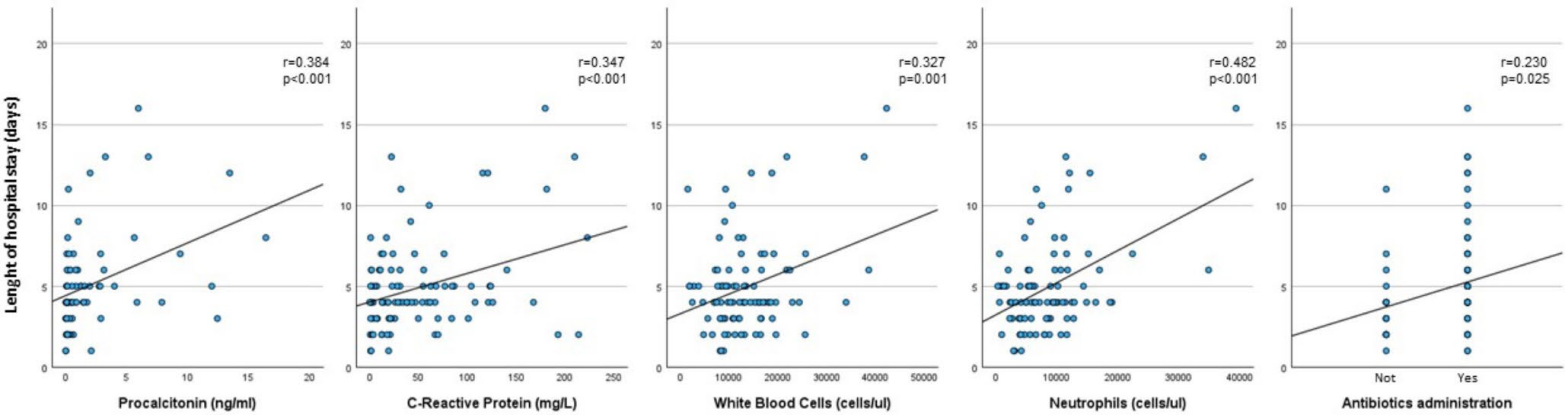
Correlation between the length of hospital stay and the elevation in leucocyte count, neutrophil count, C-Reactive Protein level, procalcitonin levels, and antibiotic administration

Pearson’s correlation was run to assess the relationship between the length of hospital stay and biochemical findings in children with HAdV infection. There was a moderate positive correlation between the length of hospital stay and the elevation in leucocyte (*r* = 0.327, *p =* 0.001) neutrophil counts (*r* = 0.482, *p <* 0.001), CRP (*r* = 0.347, *p <* 0.001) and procalcitonin levels (*r* = 0.384, *p <* 0.001) (Fig. 4). In addition, antibiotic administration was also associated with a longer hospital stay (*r* = 0.230, *p* = 0.025) (Fig. 4). The same analysis in the non-HAdV group did not reveal correlation between the length of hospital stay and the elevation in leucocyte (*r* = 0.099, *p =* 0.425) neutrophil counts (*r* = 0.071, *p* = 0.569), CRP (*r* = 0.069, *p* = 579), procalcitonin levels (*r* = 0.212, *p =* 0.252) and, antibiotic administration (*r* = 0.134, *p =* 0.274).

### HAdV coinfections

In the group of HAdV-infected children, 56/98 (57.1%) received a diagnosis of viral coinfection showing at least another respiratory virus other than HAdV. In the group of coinfected children, the most frequently observed viruses were rhinovirus/enterovirus, detected in 27 out of 56 children, and Influenza A-B, detected in 14 children (Fig. 2b).

Comparing children with HAdV infection alone and children with a viral coinfection, no differences in laboratory parameters nor in chest X-rays were detected by comparing the two groups. Also clinical manifestations did not differ between the two groups, except for wheezing, which was more frequent in children with coinfections (*p* = 0.029) with no differences between children with and without rhino/enterovirus coinfection (*p* = 0.754).

In addition, no significant differences were observed between the antibiotic administration rate. Nevertheless, oxygen supplementation, inhaled therapies, and systemic steroids were more frequent in children with viral coinfection (*p* < 0.05) (Table 4).

**Table 4:** Demographic, clinical features, biochemical and radiological findings, therapies, and outcomes of the HAdV population, comparing children with viral coinfections and children with HAdV alone. Abbreviations: HAdV, Human adenovirus; n, number; SD, standard deviation; CRP, C-reactive protein

	Viral coinfection (*n* = 56)	HAdV alone (*n* = 42)	*p*
Demographic features			
Male sex, *n* (%)	37 (66.1)	19 (51.4)	0.123
Mean age, months (SD)	38.8 (35.8)	51.7 (39.8)	0.096
Clinical features			
Fever, *n* (%)	47 (83.9)	41 (97.6)	**0.025**
Cough, *n* (%)	42 (75.0)	30 (71.4)	0.433
Respiratory distress, *n* (%)	9 (16.1)	2 (4.8)	0.073
SpO2, % (SD)	97.8 (2.1)	98.3 (1.1)	0.168
Wheezing, *n* (%)	13 (23.2)	3 (7.1)	**0.029**
Length of hospital stay, mean (SD)	5.2 (3.5)	4.9 (2.4)	0.742
Biochemical parameters, means (SD)			
WBC, cells/µL	14410 (8602)	12444 (5734)	0.207
Neutrophils, cells/µL	9072 (7970)	8180 (4834)	0.527
Lymphocytes, cells/µL	3943 (2620)	3209 (1918)	0.133
CRP, mg/L	50.3 (57.2)	51.6 (50.5)	0.909
Procalcitonin, ng/mL	2.0 (3.8)	1.7 (2.3)	0.647
Chest X-ray			
Abnormal findings	20/33 (60.6)	16/26 (61.5)	0.578
Interstitial	8/33 (24.2)	6/26 (23.1)	0.917
Lobar	12/33 (36.4)	10/26 (38.5)	0.869
Therapies			
Antibiotics, *n* (%)	46 (82.1)	30 (71.4)	0.155
Antibiotic duration, mean (SD)	5.2 (2.1)	5.3 (2.1)	0.838
Systemic corticosteroids, *n* (%)	18 (32.1)	6 (14.3)	**0.034**
Bronchodilators, *n* (%)	29 (51.8)	9 (21.4)	**0.002**
Inhaled steroids, *n* (%)	31 (55.4)	11 (26.2)	**0.003**
Oxygen supplementation, *n* (%)	8 (14.3)	1 (2.4)	**0.043**
Intravenous Immunoglobulin, *n* (%)	0	2 (4.8)	0.181

### Comparison between children with HAdV alone versus those with other viral infections

Comparing children with HAdV infection alone and those with other viral infections, excluding cases with coinfections, the results were confirmed. Fever was more common in children with HAdV alone (97.7% versus 74.0%, *p* < 0.001), who also had a longer fever duration (6.2 ± 3.9 versus 3.4 ± 2.3 days, *p* < 0.001) compared with the control group. Children with HAdV alone more commonly reported sore throat (54.5% versus 13.7%, *p* < 0.001) and lymphadenopathy (43.2% versus 12.3%, *p* = 0.02) than HAdV-negative children who, in turn, had more severe respiratory symptoms, including wheezing (23.3% versus 6.8%, *p* = 0.017), distress (45.2% versus 6.8%, *p* < 0.001) and lower oxygen saturation level (96.0 ± 3.8 versus 98.3 ± 1.2, *p* < 0.001), with a higher number of children with hypoxemia ≤ 94% (23.3% vs. 0%, *p* < 0.001). The incidence of other symptoms did not differ between groups.

Children with HAdV showed increased inflammatory markers compared to children with other respiratory infections, as demonstrated by higher CRP (54.1 ± 55.4 versus 16.5 ± 33.8 mg/L, *p* < 0.001), procalcitonin (1.7 ± 2.3 versus 0.7 ± 1.2 ng/mL, *p* = 0.032), fibrinogen (472.5 ± 131.5 versus 353.2 ± 137.0 mg/dl, *p* = 0.008) and neutrophil count (8272 ± 4745 versus 5349 ± 4003 cells/mm^3^, *p* < 0.001). In addition, no significant differences were observed in chest X-rays abnormalities (63.0% vs. 76.9%; *p* = 0.210).

The antibiotic administration rate was higher in children with HAdV alone (70.5% vs. 27.4%; *p* < 0.001), whereas oxygen supplementation was more frequent in children with viral coinfection (4.5% vs. 37.0%; *p* < 0.001).

## Discussion

HAdVs are frequently responsible for respiratory infection in pediatric age, both in immunocompetent and immunocompromised children [3], and provide clinical challenges regarding differential diagnosis and effective treatment strategies. To date, only a limited number of clinical studies [8–13] have focused explicitly on evaluating HAdV infections in the pediatric population, raising the need for further research in this area. The clinical manifestations of HAdV infections ranged from mild upper respiratory symptoms to severe lower respiratory tract infections [2]. The challenge in clinically diagnosing HAdV infections is related to their clinical overlapping with other bacterial or viral respiratory pathogens.

To the best of our knowledge, this study represents the first comprehensive analysis, in the post-COVID era, of clinical presentations, biochemical markers, radiological findings, outcomes, and management strategies for HAdV infections in a large cohort of hospitalized pediatric patients with respiratory symptoms. Notably, our comparative analysis revealed a more pronounced inflammatory response in children with HAdV infection compared to those with other respiratory pathogens.

Although HAdV infections were detected throughout the year, we found a peak incidence in spring, disagreeing with other studies. Krajden et al. [14] reported a lack of seasonal variation, while Peled et al. [8] reported a peak incidence in winter (December-February). Interestingly, this difference could be due to epidemiological changes related to the COVID-19 pandemic. The global efforts to limit the spread of COVID-19, including physical distancing, mask-wearing, school and childcare center closure, and travel restrictions, disrupted respiratory viruses circulation, resulted in a lack of exposure to classical viral infectious agents with a reduced population immunity. After the end of the pandemic limits, an increase in respiratory virus circulation led to a greater incidence of susceptible children who, during the pandemic, have not encountered this virus [15, 16].

In our cohort, children with HAdV infection were younger than children with other infections, and the majority of HAdV was detected in children younger than five years old (75%), emphasizing the vulnerability of this age group. Notably, our cohort’s patient age was slightly higher than that reported in previous studies [7, 8, 11], suggesting potential influences of regional variations, different study populations, or changes in virus circulation dynamics, particularly in the context of the COVID-19 era. The described reduction in population immunity resulting from decreased virus circulation during the pandemic may have contributed to changes in the target age of HAdV infections.

In our cohort, the prevalent clinical manifestations included fever (90%) with a mean duration of around five days, cough (75%), sore throat (52%), and lymphadenopathy (32%). While these results align with the findings of previous studies [7, 8], our study, due to its design, excluded patients with only a GI involvement, contributing to the lower representation of GI symptoms in our cohort of HAdV infection.

Children with HAdV infection experienced a higher frequency and longer duration of fever and exhibited a greater incidence of febrile seizures (15.3%) compared to those with other viral infections (6.8%). These findings confirm the results of a prospective multicenter study on the viral etiology of febrile seizures, highlighting the predominant role of HAdV among respiratory viruses in causing febrile seizures (accounting for 55.5% of total cases) [17].

Previous studies [18] identified HAdV as a potential pathogen in severely immunocompromised children, who often present with pneumonia and require oxygen supplementation. In our study, we observed a lower respiratory tract involvement with abnormal chest radiography in 35 cases (60%), wheezing in 16 cases (16%), and hypoxemia in 3 cases (3%) of HAdV patients with normal immune function. These findings closely agree with Sun et al. [7] and Yao et al. [19], where abnormal chest radiography was observed in 48% and 86% of patients, respectively. Radiographic findings did not significantly differ between children with HAdV infections and those with other respiratory viruses. This suggests that chest X-rays may have limited utility in differentiating HAdV from other viral causes of respiratory illness.

Children with HAdV infection experienced lower rates of respiratory distress, oxygen requirement, and ICU admission compared to those infected with other respiratory viruses. However, despite a lower clinical respiratory severity, HAdV-infected children had a more intense inflammatory pattern in their course, as shown by the frequency and intensity of biochemical marker alterations, offering valuable insights into the hallmarks of this viral illness. As described by other authors [7–10, 20], our study revealed significantly elevated levels of inflammatory markers, including CRP, procalcitonin, and fibrinogen, in children with HAdV compared with those with other respiratory pathogens. These findings demonstrate a more intense systemic inflammatory response in HAdV-infected individuals, similar to bacterial rather than viral infections.

As a logical consequence of the described clinical and biochemical hallmarks, children with HAdV infection were significantly more likely to receive antibiotic treatment than those with other infections, which is supported by the positive correlation between the length of hospital stay and antibiotic administration in the HAdV children. This data aligns with previous studies in pediatric populations, which reported a tendency for antibiotic overprescription in cases of HAdV infection [8, 11]. Specifically, the bacterial-like inflammatory response and the absence of specific biomarkers pointing specifically to a diagnosis of HAdV lead to antibiotic overprescription and unnecessary hospitalizations, contributing to healthcare burden and concerns for parents and physicians. This emphasizes the importance of improving diagnostic strategies to differentiate between viral and bacterial etiologies in pediatric respiratory infections, thus optimizing antibiotic stewardship practices. Implementing rapid antigen detection kits would be advisable for upper respiratory tract infections and might support the watchful waiting approach despite the elevated serum inflammatory markers [21]. Recently, a host-protein score (BV score) for differentiating bacterial from viral infection that combines the expression levels of TNF-related apoptosis-induced ligand, interferon gamma-induced protein-10, and CRP has proven helpful in differentiating between adenoviral and bacterial-adenoviral co-infection in a cohort of children with PCR-positive adenovirus detection [22], suggesting that the implementation of this tool can contribute to improve the appropriate antibiotic use.

The aberrant inflammatory response observed in HAdV children also poses significant challenges in differentiating HAdV infections from Kawasaki disease, an inflammatory condition frequently reported in the same age group [23]. In our cohort, 4% and 2% of children in the HAdV group received a diagnosis of Kawasaki disease and immunoglobulin treatments, respectively. Those two conditions share alterations in inflammatory markers and clinical features such as prolonged fever, skin rash, conjunctivitis, mucous membrane changes, and adenopathy, making differential diagnosis challenging. A link between previous Adenovirus infection and Kawasaki disease has been proposed based on extensive epidemiological data [23]. Although the exact mechanisms underlying this association remain under investigation, the study of Zsengelle´r et al. [24] suggests that the internalization of HAdV by alveolar macrophages is responsible for early proinflammatory signaling, making the virus capable of triggering an increased immune response, contributing to the development of Kawasaki-like conditions. Whether HAdV acts as a trigger for Kawasaki disease or if Kawasaki disease serves as a potential differential diagnosis for HAdV infection remains unclear.

Finally, many patients exhibited viral coinfections, affecting up to half of the HAdV group. This confirms the improved detection of viruses in children with respiratory tract infections through PCR technology advancements [25]. Remarkably, despite viral coinfections, no significant differences were observed in clinical manifestations, laboratory parameters, and radiological findings between children exclusively infected with HAdV and those with viral coinfections. This suggests that HAdV plays a predominant role in shaping the clinical and biochemical presentation and the observed inflammation, regardless of the presence of other viral pathogens. Nevertheless, a significant difference was observed in the administration of systemic steroids, inhaled bronchodilators and oxygen supplementation being required in children with HAdV and viral coinfections than HAdV alone, likely also related to the increased frequency of wheezing in those patients, suggesting a higher severity of illness, in terms of therapeutic needs, in the presence of a second viral agent, in line with the findings of a recent study on pediatric SARS-CoV-2 and respiratory coinfections [26].

The study’s strength lies in its comprehensive clinical, biochemical, and radiological analysis. However, some methodological limitations warrant consideration. While the study design allows for valuable insights into the clinical presentation and outcomes of pediatric HAdV infections, serological differentiation of HAdV serotypes/types was not performed. It is important to acknowledge that distinct clinical features observed in our study may be associated with specific HAdV serotypes/types. Additionally, the retrospective nature of the study and the inclusion of data from eight Italian hospitals raise potential concerns about selection bias and generalizability of the findings. To mitigate this, we implemented a standardized minimum set of investigations for all enrolled patients.

In conclusion, this study adds valuable insights to our understanding of HAdV infections in children. Our findings shed light on treatment practices, clinical outcomes, and the link between biochemical markers and disease severity. Interestingly, our data suggests that HAdV-infected children exhibit a more pronounced inflammatory response despite experiencing less severe respiratory symptoms compared to other viruses. Consequently, the initial challenge of differentiating HAdV from bacterial infections often leads to antibiotic overuse. Notably, fever and a strong inflammatory response are key factors driving antibiotic initiation before definitive diagnosis. Future research is crucial to develop reliable diagnostic tools, optimize treatment strategies, and ultimately improve outcomes for children with HAdV-related respiratory illnesses.

## Data Availability

The data that support the findings of this study are not openly available due to reasons of sensitivity and are available from the corresponding author upon reasonable request. Data are located in controlled access data storage at University Federico II of Naples.
